# Nature’s fight against plastic pollution: Algae for plastic biodegradation and bioplastics production

**DOI:** 10.1016/j.ese.2020.100065

**Published:** 2020-11-05

**Authors:** Wen Yi Chia, Doris Ying Ying Tang, Kuan Shiong Khoo, Andrew Ng Kay Lup, Kit Wayne Chew

**Affiliations:** a School of Energy and Chemical Engineering, Xiamen University Malaysia, Selangor Darul Ehsan, 43900, Malaysia; b College of Chemistry and Chemical Engineering, Xiamen University, Xiamen, 361005, Fujian, China; c Department of Chemical and Environmental Engineering, Faculty of Science and Engineering, University of Nottingham Malaysia, Jalan Broga, 43500, Semenyih, Selangor Darul Ehsan, Malaysia

**Keywords:** Algal polymers, Microalgae, Microplastics, Polyhydroxyalkanoate (PHA), Polyhydroxybutyrate (PHB)

## Abstract

The increased global demand for plastic materials has led to severe plastic waste pollution, particularly to the marine environment. This critical issue affects both sea life and human beings since microplastics can enter the food chain and cause several health impacts. Plastic recycling, chemical treatments, incineration and landfill are apparently not the optimum solutions for reducing plastic pollution. Hence, this review presents two newly identified environmentally friendly approaches, plastic biodegradation and bioplastic production using algae, to solve the increased global plastic waste. Algae, particularly microalgae, can degrade the plastic materials through the toxins systems or enzymes synthesized by microalgae itself while using the plastic polymers as carbon sources. Utilizing algae for plastic biodegradation has been critically reviewed in this paper to demonstrate the mechanism and how microplastics affect the algae. On the other hand, algae-derived bioplastics have identical properties and characteristics as petroleum-based plastics, while remarkably being biodegradable in nature. This review provides new insights into different methods of producing algae-based bioplastics (e.g., blending with other materials and genetic engineering), followed by the discussion on the challenges and further research direction to increase their commercial feasibility.

## Highlights

• Marine plastic pollution endangers and affects aquatic wildlife and their habitat.
• Interaction between algal enzymes and plastic polymers triggers biodegradation.
• Bioplastics can be made using protein- and carbohydrate-based polymers from algae.
• Production of polymers from algae can be enhanced through genetic engineering.
• Conventional plastics could be fully replaced by algal bioplastics in the future.

## Graphical abstract

**Figure:**
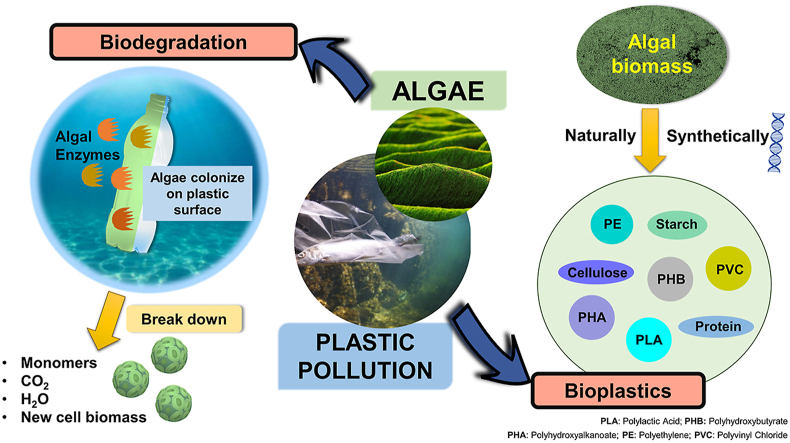

## 1 Introduction

**Fig. 1:**
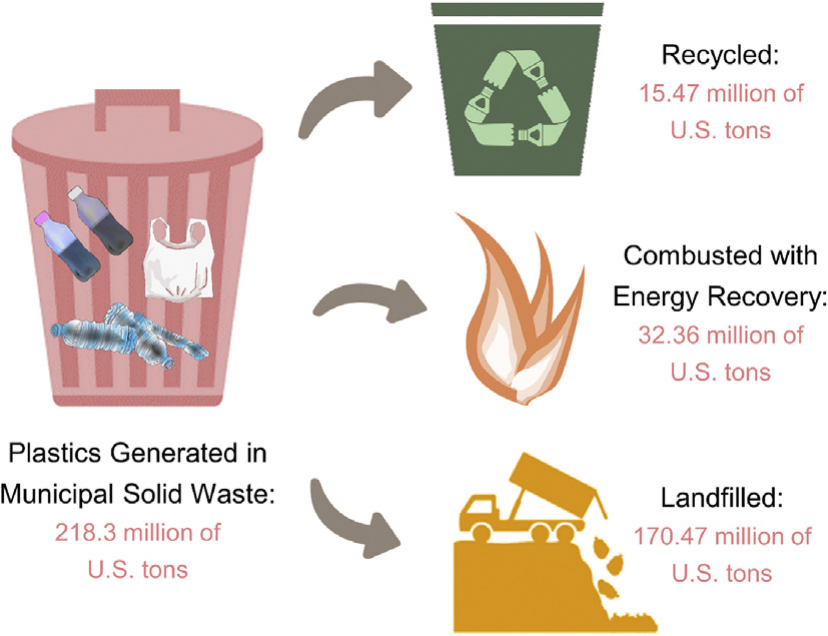
The fate of plastics generated in municipal solid waste in the United States from 1960 to 2017. Data is retrieved from Ref. [3].

Global production of plastics had increased to around 359 million metric tons in 2018, from 245 million metric tons in 2008, and it is expected to be tripled by the year of 2050, accounting for a fifth of global oil consumption [1]. Despite the mass production of plastics since the 1950s, there is yet effective strategy implemented to tackle the disposal issues brought by plastic waste. As shown in Fig. 1, the recycling rate of plastics is relatively low compared to the plastics generated while the majority of them are being disposed in the landfills. However, the decomposition of plastics is the hardest among all the general commodities such as fruits, papers, leathers and aluminium. This is because it may persist in the nature for centuries before decaying [2].

This “white pollution” caused by tremendous plastic debris has also occurred in the aquatic environments, causing serious impacts on marine life such as ocean animals and coral reefs. For instance, these marine debris has caused problems such as ingestion, entanglement, debilitation and suffocation to the marine species, leading to reduced life quality, drowning, limited capabilities to avoid predator, lower reproductive capacity, impairment of feeding ability and death [4]. There are also increasing concerns on the potential effects of microplastics on human health since microplastics have been detected in food and air samples. With all the severe consequences, methods to help in plastic degradation and alternatives to conventional plastics have attracted the attention of researchers since the use of plastic materials are inevitable for daily life needs.

Plastic can be reduced to its basic constituents in a rapid manner with the help of biological agents. Various microorganisms have great potential to convert certain plastic polymer biologically into simpler products via aerobic and anaerobic mechanism [5]. For example, a biological agent could utilize the organic polymer as a nutritional substrate for energy and growth, resulting in microbial biomass as the end product of complete biodegradation [6]. Recently, it was found that various microalgae promote biodegradation of polymers and the energy needed for degradation is reduced, since the synthesized enzymes with simple or multiple toxin systems involve a reduction in activation energy to weaken the chemical bonds in the polymer [7].

On the other hand, there is an increasing demand on the environmental-friendly polymers (i.e., biodegradable plastics) in various applications such as packaging, health and agriculture industry. Nowadays, terrestrial crops such as potatoes and corn are used to derive bioplastics and this leads to competition with food supplies as well as consumption of large land areas, water and nutrients, making this kind of bioplastic production not sustainable for long term [8,9]. This makes it important to synthesize new bio-based biodegradable plastic polymers by developing an in-depth understanding of biomass structure which is able to manufacture new biodegradable plastics [5].

Microalgae which are able to grow on waste resources with high lipid accumulation can be a potentially better candidate as a source of biomass for bioplastic production as it does not compete with food sources [10,11]. In addition, microalgal biomass presents potential of mass production and greenhouse gas uptake due to their rapid growth and high carbon fixing efficiency when they grow [[12], [13], [14]]. Approaches to produce microalgal bioplastics include (1) composites produced by blending microalgal biomass, petroleum- or bio-based polymers and additives as well as (2) biopolymers like polyhydroxybutyrates (PHBs) and starch cultivated intracellularly within microalgal cells [15].

As of our best knowledge, plastic degradation and production using algae are scarcely reviewed in the literature. This review study discusses their current status and progress by elaborating on the background study of plastics and the associated marine pollution to emphasize the urgent need of developing environmentally friendly techniques and products. Mechanism of biodegradation is presented with identification of potential algae species followed by the discussion on how microplastics affect algae. Subsequently, the potential of algae as a source of bioplastics is comprehensively reviewed, including blending microalgae with other materials and genetic engineering for microalgae strains which produce biopolymer. This review has also highlighted the research challenges and future prospects to provide valuable insights into the relationship between microalgae and plastics.

## 2 Marine plastic pollution

Marine ecosystem plays a vital role in supplying the world wealth of ecosystem services such as food security, carbon storage, waste detoxification and cultural benefits (e.g., recreational opportunities and spiritual enhancement) [16]. Human threats such as disposal of plastic waste directly or indirectly into the marine ecosystem have negatively impacted the wellbeing of both human and marine ecosystem [17,18]. The term “marine plastic pollution” was defined owing to a substantial and increasing amount of plastic waste exposure in the marine ecosystem [19]. The perpetual of plastics waste entering the world’s oceans from land-based sources will increase tremendously within the next decade [20]. In fact, as time goes by, these plastic wastes may fragment into smaller pieces of around 0.1 μm–5 mm (i.e., microplastics) scattered in the environment over geological timescales, leading to the need of time intensive, high cost and laborious management in removing these marine plastics.

### 2.1 Ecological, social and economic impacts of plastic waste

The ubiquity of plastic waste is essentially irreversible to be removed completely in the marine ecosystem as the pollution will always exceed this critical condition, making ocean as the ultimate grave of most mismanaged plastics. The ecological impacts occurring globally were ingestion and entanglement on tens of thousands of individual fishes, turtles, birds and mammals [21]. Besides that, the marine debris also damaged the habitats and organisms including shorelines, coral reefs, shallow bays, estuaries, open ocean and deep sea [[22], [23], [24], [25], [26]]. There is a widespread of mainstream media that portrays collapsed beached whales, turtles and seabirds with stomachs full of plastic waste, showing the severity of how these wastes have impacted the marine organisms. These charismatic marine species retain a significant contribution towards the society and if these crisis were not overcomed, future generations might never have a direct chance to observe the existence of these marine animals [27,28].

The marine plastics are frequently ingested by marine species and this may impact the food chain of fish and shellfish stocks, and their prey in diminishing their reproduction, growth and population level [17]. Eventually, these contaminated marine species may be consumed by social communities as seafood and this indirectly affects the risk of human health. The consumption of toxic persistent organic pollution in the composition of polymers (i.e., plasticizers, biocides and flame retardants) poses an additional risk to the social communities [29]. The fishing and aquaculture industry is highly fragile as the productivity, viability, profitability and safety are affected by plastic waste present in the marine ecosystem [29].

Despite that, the ecosystem service has reported the substantial negative impact of plastic waste on experiential recreation where the coastlines are exposed to plastic. This directly contributes to the economic costs impacted by these plastic wastes especially in the cleaning up process for the ecosystem [30]. The presence of plastic wastes on the shore may be one of the main key reasons why visitors will spend less time in these environments or avoid certain sites, decreasing the economics of tourism revenue [31]. Other direct consequences caused by the presence of plastic wastes are a range of unexpected injuries (e.g., sharp debris, entangled in nets and exposure to unsanitary items), detrimental to physical and mental wellbeing (e.g., social interaction, family bonds and physical activity) and intensively colonized by a wide range of opportunistic species [32,33].

## 3 Overview of plastics

Plastics are synthetic organic polymers, which are hydrophobic, inert, high-molecular-weight long chains of molecules (monomers) that joined together by covalent bonds [34]. Their properties include malleable, mouldable, strong, durable, lightweight and inexpensive, making them suitable for the manufacturing of a variety of products including household items, product packaging and shopping bags, which are mostly single-use products.

Traditionally, synthetic plastics are produced using refined petroleum products where the synthetic polymers made up of carbon-carbon bonds are derived in a controlled environment [35]. These conventional plastics made of heavy crude oil could lead to issues of fossil resource depletion, climate change and greenhouse gases emissions [36]. However, the biodegradability of plastics relies on their chemical structure but not on the sources used to collect the monomers (Siracusa and Blanco, 2020). For example, commonly used plastics, polyethylene terephthalate (PET), polyvinyl chloride (PVC), high-density polyethylene (HDPE), low-density polyethylene (LDPE), polystyrene (PS), polypropylene (PP), and miscellaneous plastics are non-degradable plastics even though the starting monomers of polymers such as PE, PP, PVC and PET could be obtained from biological resources. These plastics are composed of high molecular weight due to their repetitions of small monomer units [37,38].

Degradable plastics can be divided into four groups: compostable plastics, photodegradable plastics, bio-based plastics and biodegradable plastics. Biodegradable plastics, which usually break down while interacting with water, enzymes, UV and gradual changes in pH, can be produced by renewable resources including components of animals, living plants and algae as well as microorganisms. An example of biodegradable plastics is polyhydroxyalkanoate (PHA) which is completely biodegradable and has similar properties as conventional plastics. This biodegradable bioplastic approach is extremely resource-efficient with the advantages of saving energy, avoiding food waste and reducing carbon dioxide emissions [39].

### 3.1 Plastic waste treatment

Conventional methods to treat plastic waste include (1) primary method: re-introduce plastic scrap in the heating cycle of plastic processing line itself for higher production, (2) secondary method: mechanically re-extrude, process and converting plastic waste into new plastic products blended with virgin polymers for overall production cost reduction, (3) tertiary method: chemically or thermo-chemically alter the polymer structure of plastic to employ it as monomer feedstock in industrial recycling loops [40]. These methods are basically plastic recycling, but not all plastics can be recycled. For instance, there are two different types of plastics, thermoplastics and thermosetting polymers. The latter contains polymers which cross-link to form an irreversible chemical bond and cannot be re-melted into new material regardless of the amount of heat applied [41]. In addition, recycling of plastics is relatively inefficient and less cost-effective where it deteriorates the quality of the polymers yielded [6]. Therefore, biodegradation of plastic is an alternative effective, eco-friendly and innovative method.

Biodegradation refers to any chemical or physical change in material resulted from biological activity. Generally, a biological agent utilizes organic polymer as a substrate for their energy and growth, resulting in microbial biomass as the end product of complete biodegradation. For example, *Ideonella sakaiensis* 201-F6 was observed to consume PET as the sole carbon source. Two novel enzymes (i.e., PET hydrolase (PETase) and mono(2-hydroxyethyl) terephthalic acid hydrolase (MHETase)) were discovered to hydrolyze PET into non-toxic monomers (e.g., terephthalic acid (TPA), ethylene glycol (EG)) [42]. Besides *I. sakaiensis* which naturally secretes PETase, several bacterial systems such as *Bacillus* and *Escherichia* have been tested to produce synthetic PETase [43]. At least 27 enzymes which degrade synthetic oligomers or polymers have been identified and most of them were cutinase, lipase and esterase [44,45]. In most cases, multiple enzymes and metabolic pathways must be employed for plastic degradation instead of a single enzyme [46].

### 3.2 Plastic biodegradation by algae and mechanism

A possible approach to mitigate the “white pollution” is to identify potential algae and its toxins which could effectively break down the polymeric materials biologically [7]. Comparing to the bacterial systems which may be considered as a biological pollutant due to endotoxins and requirement of a rich carbon source for growth, microalgae is a potential candidate since they do not contain endotoxins and organic carbon sources are not required under photoautotrophic conditions [47]. Moreover, *I. sakaiensis* and other microbes used for PETase generation do not adapt well to marine habitats where the accumulation of most plastic waste occurs [48].

Algae are known to colonize on artificial substrata like polythene surfaces in sewage water (Table 1) and these colonizing algae were found to be less hazardous and non-toxic [49]. Adhesion of algae on the surface will initiate the biodegradation and their production of ligninolytic and exopolysaccharide enzymes is the key for plastic biodegradation [50]. The algal enzymes present in the liquid media interact with macromolecules present at the plastic surface and triggers the biodegradation [51]. The polymer is utilized by algae as carbon source since the species growing on the PE surface were to found to have higher cellular contents (protein and carbohydrates) and higher specific growth rate [52].

**Table 1:** Colonization of algae on plastic surface.

Algae species	Plastic	Water Body	Reference
*Scenedesmus dimorphus* (green alga)*, Anabaena spiroides* (blue-green alga) *and Navicula pupula* (diatom)	Dumped waste polyethylene bags	Domestic wastewater in Chennai City, Tamil Nadu, India	[6]
*Oscillatoria princeps, O. acuminate, O. subbrevis, O. willei, O. amoena, O. splendida, O. vizagapatensis, O. limnetica, O. earlei, O. peronata, O. formosa, O. okeni, O. geitleriana, O. limosa, O. chalybea, O. salina, O. rubescens, O.curviceps, O.tenuis* and *O. laete-virens*	Submerged polythene	Domestic sewage water bodies of Silchar town, Assam	[57]
*O. limnetica, P. lucidum, Phormidium calcicola, O. earlei, Lyngbya cinerascens, Nostoc carneum, Nostoc linckia, Spirulina major, Hydrocoleum sp., Chlorella sp., Pithophora sp., Scenedesmus quadricauda, Calothrix fusca, Stigeoclonium tenue, Calothrix marchica, Anomoeoneis sp., Oedogonium sp., Arthrospira platensis, Navicula minuta, Nitzschia sp., Navicula dicephala, Nitzschia intermedia, Spirogyra sp.* and *Synedra tabulata*	Polythene carry bags	Solid domestic sewage dumping sites	[50]
*Phormidium lucidum, Oscillatoria subbrevis, Lyngbya diguetii, Nostoc carneum* and *Cylindrospermum muscicola*	Dumped waste polythene bags	Domestic sewage water drains of Silchar town, Assam	[58]
*Phormidium tenue, Oscillatoria tenuis, Monoraphidium contortum, Microcystis aeruginosa, Closterium constatum, Chlorella vulgaris,* and *Amphora ovalis*	Waste polythene materials	Various ponds, lakes, and water bodies of Kota city in the state of Rajasthan, India	[49]
*Coleochaete scutata, Coleochaete soluta, Chaetophora, Aphanochaete, Gloeotaenium, Oedogonium, Oocystis, Oscillatoria, Phormidium, Chroococcus, Aphanothece, Fragilaria, Cocconis, Navicula,* and *Cymbella*	Polythene	Oligotrophic water bodies of Lucknow, Uttar Pradesh	[59]

Additionally, surface degradation or breakdown have be seen clearly on the transverse section of the algal-colonized PE sheets [6]. Five methods of biodegradation including fouling, corrosion, hydrolysis and penetration, degradation of leaching components as well as pigment coloration via diffusion into the polymers were observed in past works. Blue-green alga (Cyanobacterium), *Anabaena spiroides,* showed the highest percentage of LDPE degradation (8.18%) followed by diatom *Navicula pupula* (4.44%) and green alga *Scenedesmus dimorphus* (3.74%) [6]. A study by Sarmah and Rout [52] also concluded that freshwater nontoxic cyanobacteria (*Phormidium lucidum* and *Oscillatoria subbrevis*) which are readily available, fast-growing and easily isolable, are capable of colonizing the PE surface and biodegrading LDPE efficiently without any pre-treatment or pro-oxidant additives.

Besides that, Gulnaz and Dincer [53] investigated biodegradation of bisphenol A (BPA), which is a widely used polymer in plastic industry, using *Aeromonas hydrophilia* bacteria and *Chlorella vulgaris* microalgae. The results indicated that the BPA was easily degraded by algae and its concentrations were below detection limits after 168 h without estrogenic activities [53]. Similar results were obtained in a study by Hirooka et al. [54] that BPA was degraded to compounds without estrogenic activity using green alga *Chlorella fusca* var. *vacuolata.*

Apart from that, microalgae can be genetically modified to a microbial cell factory which is capable of producing and secreting plastic degrading enzymes. For example, green microalgae *Chlamydomonas reinhardtii* was transformed to express PETase and the cell lysate of the transformant was co-incubated with PET, resulting in dents and holes on the PET film surface as well as TPA, which is the fully degraded form of the PET [55]. Moog, et al. [56] also successfully used *P. tricornutum* as a chassis to produce PETase which showed catalytic activity against PET and the copolymer polyethylene terephthalate glycol (PETG). These studies have provided a promising environmentally friendly solution to biologically degrade PET using microalgae via synthetic biology.

### 3.3 Effect of microplastic on algae

Microplastic with size <5 mm either results from abiotically degraded macroplastics or specially manufactured products (e.g., drugs or personal care products). These microplastic could be detected nearly everywhere and further degraded into smaller particles, i.e., nanoplastics which have size <100 μm. Waller et al. [60] reported that even areas with the least population and highest inaccessibility, such as the Antarctic region, is also contaminated with microplastics. Microplastic particles contain harmful additives and can absorb hazardous compounds such as organic pollutants and heavy metals as well as invade the food chain at the level of microorganisms or small animals due to their tiny size and physical properties [61].

In a recent study by Taipale et al. [62]; mixotrophic algae (*Cryptomonas* sp.) feeding on microbiome colonized on PE microplastic, where it sequestered carbon in the polyethylene microplastic (PE-MP) to synthesize essential ω-6 and ω-3 polyunsaturated fatty acids (PUFA). It was found that the microbes colonizing on the microplastic resulted in higher growth rates of the algae compared to the control treatment. However, direct contact to the PE-MP or its releasing chemicals has a toxicological impact on mixotrophic algae (*Cryptomonas* sp.) since there was an absence of microbes utilizing the chemicals covering the plastic surface [62].

Investigating the influence of microplastics on the growth of *Spirulina* sp., Khoironi and Anggoro [63] reported that the higher the concentration of microplastics, the lower the growth rate of the microalgae. This is because the presence of microplastics in culture may cause shading effects which lead to reduced light intensity and affect microalgal photosynthesis [64]. However, in a research by Zhang et al. [65]; the negative impact of microplastic on microalgae was not because of the shading effect, but the interaction between microalgae and microplastic such as aggregation and adsorption. This explains that the effects of microplastic on microalgae depends on the particle size of microplastic [65]. According to Liu et al. [66]; microplastic with larger size caused serious impacts by blocking the transport of light and affecting photosynthesis while microplastic with smaller size destroyed the microalgal cell wall by absorbing onto its surface.

On the contrary, Canniff and Hoang [67] reported that *Raphidocelis subcapitata* experienced higher growth in exposure media containing plastic microbeads (63–75 μm) than the control. In addition, findings of Chae et al. [68] showed that cell growth and photosynthetic activity of marine microalga *Dunaliella salina* were promoted without influence on cell morphology when exploring the impact of microplastics, which were larger than the algal cells (about 200 μm diameter). The promoted growth was likely due to the trace concentrations of additive chemicals which could be leached from microplastics such as stabilizers, phthalates and endocrine disruptors [68]. However, there should be an investigation on whether algae utilize microplastic as a carbon source for growth, like using plastic, as discussed in Section 3.2. In brief, more studies are needed focusing on how microplastics impact microalgae which play a crucial role as primary producers of ecosystems. This is important in order to investigate the potential of microalgae biodegrading microplastic, which has not been studied before.

## 4 Potential of algae as a source of bioplastics

Bioplastics are defined as plastics that are made fully or partially from biomass or renewable sources, such as food crops, and have the identical function as the petroleum-based plastics [69]. Bioplastics can be made up of different materials which have different properties. Generally, bioplastics are divided into three main groups [70]:

i. Bio-based but non-compostable plastics: PE, PP, PET, polytrimethylene terephthalate (PTT) or polyester elastomers (TPC-ET)
ii. Bio-based and degradable plastics: Polylactic acid (PLA), PHA, starch, cellulose
iii. Fossil resource-based plastics that are biodegradable: Polybutylene adipate terephthalate (PBAT)

**Fig. 2:**
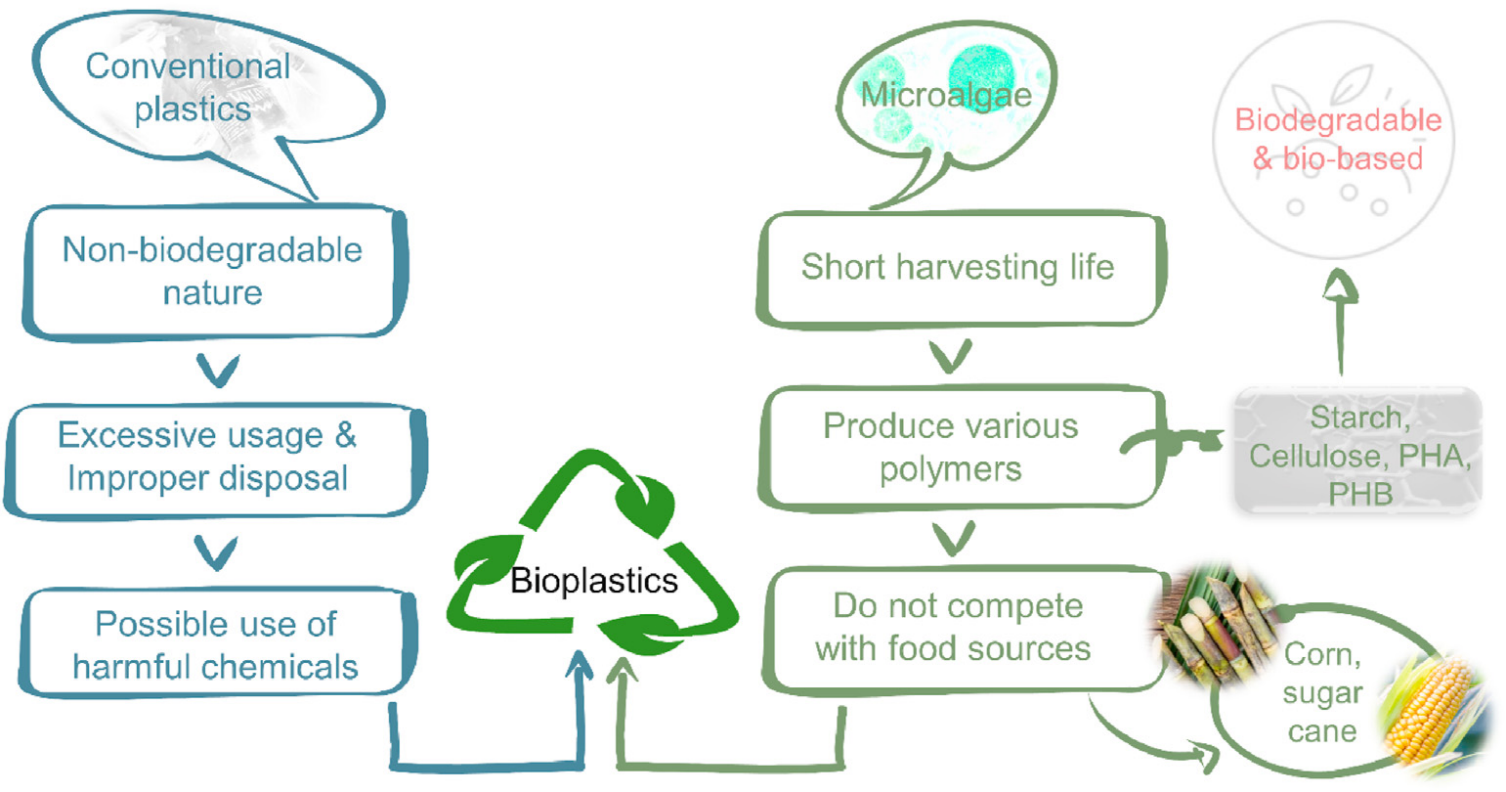
Potential of microalgae in producing bioplastics.

There are numerous sources that can be used to manufacture bioplastics, mainly agricultural crop-based crops, such as corn, wheat, soy proteins, milk proteins, collagen and gelatin. However, this raises the concern on the sustainability of these feedstocks, such as the competition between land and water resources for human consumption [71]. Furthermore, the process of extracting compounds, especially polymers from plants for the synthesis of bioplastics is difficult due to the presence of layered cell walls [72]. In addition, the “green” plastics made from food crops, such as cassava, corn or sago, face the issues of poor water resistance and mechanical properties [73]. Therefore, algae have been emerging as a novel and potential biomass source to manufacture bioplastics since algae can be cultivated on non-arable lands and have short harvesting time, as demonstrated in Fig. 2 [74,75].

Besides, algae do not compete with the food production for human consumption, are tolerant to harsh environmental conditions, can remediate wastewater and utilize carbon dioxide as a nutrient source for biomass production [76]. During the manufacturing of algae-based plastic, the encapsulation of non-biodegradable polymer, for instance polyolefin, in the thermoplastic algal blends can capture and store carbon dioxide in biomass form permanently. Consequently, carbon dioxide will not emit back into the atmosphere [77], thus alleviating the greenhouse effect. Summing up, algal-based bioplastics serve as a promising and non-toxic alternative that can reduce the use of fossil fuels, enhance plastic quality and minimize negative environmental impacts brought by the excessive use of petroleum-based plastics [78].

Microalgae-based bioplastics can be produced through a few approaches, such as direct use of microalgae biomass, blending with other materials, intermediate biorefinery processing and genetic engineering to create ideal polymer-producing microalgae strains [8]. The algal biomass comprises of protein and carbohydrate-based polymers which can be utilized as one of the bioplastics components. Currently, starch, cellulose, PHA, PHB, PLA, PE, PVC and protein-based polymers are some of the examples of the compounds from algae biomass used to develop biodegradable plastics [79]. Among these polymers, PHA is the most recommended to produce bioplastics because it can be biodegraded by enzymatic action. On top of that, PHB, a type of PHA, recently has emerged as a new polymer to produce bioplastics due to the good barrier for oxygen. The bacteria, for instance, *Ralstonia eutropha* and *Bacillus megaterium*, are known to produce PHB as an intracellular carbon source [80,81].

There have been some examples of manufactured bioplastics by the designers or the researchers [82]. For instance, Eric Klarenbeek and Maartje Dros, the designers from Dutch created a bioplastic product made from algae that could fully replace the traditional plastics. They also established AlgaeLab to cultivate algae to produce starch as a raw material for the bioplastic. Furthermore, Austeja Platukyte developed biodegradable materials from algae, consisting of agar from algae and coated calcium carbonate, that could replace petroleum-based plastics. Although the end-products are lightweight, they are waterproof, strong and durable. Moreover, the bioplastics can be composted or used as fertilizer to help maintaining soil moisture. Similarly, Ari Jónsson mixed red algae powder with water to create an alternative bottle to the traditional plastic bottle. The bottle will retain in its form when it is full of water, but it will begin to decompose when empty. The liquids kept in the bottle are safe to drink and the consumers can snack on the bottle itself as well. In short, algae act as a promising substituting feedstock for bioplastics production to replace the conventional petroleum plastics, thus solving the ocean plastic pollution.

### 4.1 Blending with other materials

On the other hand, the microalgae biomass can be blended with other materials, for example with petroleum plastics, natural products (cellulose or starch) or polymers, while manufacturing bioplastics in order to prolong their lifespan, improve their properties and enhance their mechanical performance [8]. The blending materials can be obtained from algae biomass, for instance, PLA, PHA, cellulose, starch and protein. A study by Shi et al. [77] reported that the mechanical properties of the microalgae-containing plastic films were found comparable to the plastic films (polyurethane or PE) despite the disadvantage of low tensile strength, particularly the elongation at break. Apart from that, a study by Otsuki et al. [83] used maleic anhydride to modify PE for better interaction because there was no affinity between *Chlorella* sp. and PE. The tensile strength and thermal plasticity of the composite made up of *Chlorella* sp. and modified PE were greater and more satisfactory as compared to the composite made from unmodified PE. The composite also was able to be easily molded into different shapes by heat-pressurizing processing. Therefore, blending the algae-based plastic products with other materials can improve the physical and chemical properties of the bioplastics.

Moreover, Chiellini, et al. [84] blended polyvinyl alcohol (PVA), starch with algae, *Ulva armoricana* to synthesize bioplastics. The starch reduced the amount needed of PVA by approximately 40% while at the same time, maintaining the cohesion. The film-forming properties and mechanical characteristics also showed satisfactory results. At the same time, the degradation rate of blended product made from *U. armoricana* in forest-sandy soil attests was fast, which was over 80% mineralization in 100 days, showing the potential of this resource as the biodegradable and eco-compatible composite since it will eventually be disposed of in the soil, liquid and other solid media. Shi et al. [77] also demonstrated the production of plastic materials through the combined use of plant polymer, starch with microalgae biomass while Zeller et al. [9] focused on the thermomechanical polymerization of *Chlorella* sp. and *Spirulina* sp. biomass for the synthesis of algal-based bioplastics and their thermoplastic mixtures. According to the experimental results, as compared to *Spirulina* sp., *Chlorella* sp. displayed a better bioplastic behavior but lower blending performance.

Additionally, Jang et al. [85] utilized seaweed, *Laminaria japonica* (brown algae) and *Enteromorpha crinite* (green algae), that was derived from the residues obtained during the production of bioenergy, to synthesize seaweed reinforced PP biocomposites. The study showed that *E. crinite* which was stable at high temperature was more suitable as the reinforcement of biocomposites than *L. japonica*. The strength and thermomechanical properties of green-algae-reinforced PP biocomposites were satisfactory as compared to the brown-algae-reinforced PP biocomposites. Besides, Bulota and Budtova [86] synthesized PLA biocomposites that mixed with red, brown or green algae. When the algae concentration increased, the mechanical properties of composites decreased, excluding the composites of *E. crinite* which had large particles size. It was found that the particles size of algae affected the mechanical properties of the composites where large particles demonstrated good mechanical properties. Additional research should concentrate on improving the adhesion between matrix and filler, thus tailoring the type of algae and particle size to optimize the efficiency of the composite. A work by Mathiot et al. [87] utilized microalgae to manufacture starch-based bioplastics. Among the ten microalgae strains, *C. reinhardtii* 11–32A strain was employed, followed by direct plasticization with glycerol. The starch-based bioplastics were found to have satisfactory plasticization capacity.

Machmud et al. [73] utilized the red algae, *Eucheuma cottonii*, as the raw material for plastics production via filtration technique. To produce bioplastics, red algae were mixed separately with latex of *Artocarpus altilis* and *Calostropis gigantea* to substitute the use of glycerol as plasticizer because the increased concentration of glycerol reduced the thickness and density of the bioplastics. The tensile test conducted at room temperature revealed that glycerol did not improve the ductility but simultaneously reduce the tensile strength and energy absorption of the bioplastics. On the other hand, the latex of tropical plants, *A. altilis* and *C. gigantea* did not affect the physical properties and increase the ductility, tensile strength or energy absorption of the red algae-derived bioplastics. The ultimate strength of the red algae-latex derived bioplastics was higher than starch-based plastics or other algal bioplastics blended with latex of other plants, suggesting the potential of mixing of tropical plants’ latex with algae to synthesize bioplastics that could be tailored to meet any specific requirements in various applications. In summary, the manufacturing of bioplastics aided by blending with the compounds extracted from microalgae biomass or plants can help to enhance the mechanical properties of the end-products. However, it is essential to take note that during the selection of blending materials or compounds, the compounds must be biodegradable to avoid negative environmental impact and the resulting waste management cost and pollution.

### 4.2 Genetic engineering

Genetic engineering is found to be a promising way to modify the algae strains to synthesize compounds for bioplastics production, such as PHB, a thermoplastics and biodegradable polyester produced by bacteria [80]. Through genetic engineering, the PHB production by algal can be made feasible. Applying genetic engineering by inserting the bacterial PHB production into microalgae or macroalgae could reduce the production cost as the bacterial fermentation system for producing PHB is costly for bioplastics manufacturing [80,81]. For example, Chaogang et al. [88] modified the *C. reinhardtii* strain with two expression vectors containing phbB and phbC genes from *R. eutropha* to produce PHB. The result was satisfactory through the presence of PHB granules in the cytoplasm of transgenic algal cells. This favors the research focus on increased production of PHB in the transgenic algae and accumulation of PHB in the chloroplast. Apart from that, Hempel et al. [80] introduced the bacterial PHB biosynthesis pathway into the cytosolic compartment of diatom *Phaeodactylum tricornutum*. This approach is low-cost and environmentally friendly and can achieve PHB levels of up to 10.6% of algal dry weight while accumulating them in a granule-like form in the cell cytosol.

Hence, genetic engineering is the promising approach to modify the genes of particular algae strain to enhance the production of the compounds of interest, for instance, polymers or starch, in a shorter time to synthesize bioplastics [90]. Algae are genetically simpler as compared to the plants, eukaryotic cells and other complex organisms [91,92], easing the genetic engineering procedure. However, the process of genetic modification requires the screening of thousands of mutants with desired phenotypes followed by the isolation of the new modified strain. This screening process is time-consuming to select and isolate the desired mutants. The cost of the genetic engineering is also expensive as it requires specialized equipment, genetic engineering kits and the application of the genetically modified organism (GMO) status of the newly developed strains.

Another issue with genetic engineering is the stability and suitability of the genetically modified algae strains for large scale cultivation which has more diverse conditions because the genetic modified strain is developed and tested in the precise laboratory stage. For the large scale cultivation, genetically modified algae strain will experience the variations in temperature, light intensity, pH and will be exposed to the contamination risk [92]. Another concern is the safety and ethical aspect of the genetic modified algae strain. It may pose a risk to the ecosystems, including humans, animals, and plants. There is a possibility of gene migration, at which foreign genes are spread to other plants, if the GMO is released to the environment or grown outdoor. Furthermore, genetically modified algae may cause allergic reactions in some people as plastics are a common source of occupational skin irritation or allergic contact dermatitis during the production process [93].

Overall, genetic engineering is indeed highly potential by modifying the genes of the algae strain to boost up the production of polymers or other biomolecules for the synthesis of bioplastics in spite of the issues that may arise due to genetic modification process. Therefore, it is advisable to test the genetically modified algae strains under the experimental conditions which can reflect various real situations in the field settings.

### 4.3 Challenges of algal bioplastics

Despite the overwhelming results that showed by algae-based bioplastics in laboratory scale, the commercialization and production of algae-based plastics on a large scale are still hindered by a few challenges. Firstly, the identification of the most suitable algae to produce polymers for bioplastics with different properties is necessary and essential. The algae biomass composition varies among the species. For instance, Johnsson and Steuer [94] extracted starch and PHAs to produce plastic films using ultrasonification and water from *Scenedesmus almeriensis*, *Neochloris oleoabundans* and *Calothrix scytonemicola*. Among these organisms, the PHA-rich algae, *C. scytonemicola* gave promising results. The extraction of starch from *S. almeriensis* was successful but vice-versa for *N. oleoabundans*. Nevertheless, the plastic materials produced from *N. oleoabundans* was satisfactory and further work could be carried out by synthesizing the material comprising of a mixture of different polysaccharides classes, providing a possible transformation from petroleum-based plastic to biodegradable plastics. On the other hand, Abdo and Ali [95] identified the ability of a few microalgae strains to synthesize PHB. The findings suggested that, *Microcystis aeruginosa* had the highest concentration of PHB (0.49 ± 0.5 mg mL^−1^) among the tested microalgae strains and can be used for producing bioplastic with the demonstrated good plasticizing capacity.

During the designing of bioplastics, the selection of appropriate polymers extracted from algae is also a challenge to produce sustainable bioplastics with good resilience and strength as compared to the conventional plastics. There are a few factors need to be considered during the selection of appropriate polymers such as the biodegradability, feedstock renewability, degradation rate, brittleness, consumer acceptability, polymer size, molecular weight and moisture content [96]. A number of studies have shown that the degradation of bioplastics has severe implications for the environment. The release of the greenhouse gases such as carbon dioxide and methane during the degradation or decomposition of bioplastics has raised an issue. Therefore, during the manufacturing process of bioplastics, it is essential to consider the impacts to the environment when the bioplastics are being degraded, for example, specific degradation conditions, slow disintegration process or accumulation of the released methane and other harmful gas. It is also cruicial to ensure that the bioplastics can be degraded in any environmental conditions and not release any harmful gaseous [81].

Besides, a study by Beckstrom [97] discovered the presence of unpleasant odors in bioplastics made from algae lipid as well as the slow production process due to the agglomeration tendencies caused by the carbohydrates in the algae biomass. The issue of odor was also discussed in a study by Wang et al. [89] when they modified microalgae, *Nannochloropsis* sp. and planktonic algae, catfish algae to blend with PE or PP. The plastic products synthesized from microalgae that contained high levels of fatty acids, especially polyunsaturated fatty acids, blended with PE had a foul-smelling odor as compared to catfish algae blended with PP. Absorbents like activated carbon or zeolite will then be needed to remove or alleviate the odor as this will affect the commercialization of algal-based bioplastics significantly. Apart from that, a report from Blesin et al. [98] also mentioned that there is a lack of awareness among the consumers about the use of bioplastics or they are unfamiliar with the term “bioplastics”. Some of the consumers assume that the bioplastics are expected to be more expensive due to the use of organic substances.

Algae cultivation system is another challenge that needs to be overcome before the commercialization of algal-based plastics. Large-scale cultivation is needed to mass cultivate the algae to produce polymers or other compounds, such as starch. The algae can be mass cultivated in the open pond or closed photobioreactor systems at which these two cultivation systems have its own advantages and disadvantages. Although the operating costs for open system are low and easy to maintain and scale-up, open systems face the issues of low productivity, high contamination risk and infeasibility for large-scale cultivation of specific strains. On the other hand, a closed photobioreactor system is less susceptible to the contamination, favors the growth of selected algae species and has high productivity due to high photosynthetic rate. However, the scale-up cost is expensive as compared to the open pond system [75,[99], [100], [101]]. From the analysis by Abdul-Latif et al. [36], the Sabah coastline which is approximately 3885 km^2^ could theoretically generate 13.986 Mt/y of seaweed by 2050. In view of the projected amount of algae-based bioplastic in 2050, the macroalgae cultivation requires an area of more than 0.6 million hectares to synthesize compounds for bioplastic development as well as achieve 100% reduction in emission of carbon dioxide.

Furthermore, the waste management of bioplastics is essential. There are a few methods that can be applied to waste treatment of bioplastics including mechanical and chemical recycling, incineration, composting, anaerobic digestion and landfilling. Among these methods, composting is the most suitable method for waste management of bioplastics because the biodegradable bioplastics will degrade within a short time as compared to conventional plastics. For example, PLA biodegrades in an average of 4 to 6 weeks in industrial composting facilities [102,103]. To summarize, there are some challenges faced towards the commercialization of bioplastics such as the cultivation of algae and the selection of suitable polymers or algae strains for the bioplastics production. These difficulties need to be overcome to prevent the worsening the plastic pollution and depletion of fossil fuels.

## 5 Future prospects

Microalgae contribute to the biodegradation of plastic waste with its enzymes that weaken the chemical bonds of plastic polymers. The attempt of using microalgae to convert these plastics into metabolites such as carbon dioxide, water and new biomass is of great interest. However, this field is relatively new and more studies are required. Considering the variety of plastics investigated, more biodegradation studies employing algae must be carried out and the efficiency must be evaluated. Moreover, it has yet to be researched on how microalgae degrade microplastics. More extensive research on how microplastics affect microalgae will highly benefit the understanding of the effects of its particle size on the biodegradation study. It is a huge research gap on whether microplastics will kill microalgae or the opposite where microalgae degrade microplastics since the existing researches are not conclusive. Another serious issue is that microalgae which contains hydrocarbon, proteins, lipids and other high value added compounds are mostly used for food products and must be free from pollutants, including microplastics [100]. It is essential to investigate the implications of microplastics on the health of aquatic organisms and human. With the concern on its harmful health effects, purifications of microalgal products must be carried out to ensure the safety of microalgae which are for ingestion purposes [104]. Plastic wastes of all sizes should be investigated for a better understanding in terms of the health and environmental impacts.

The study by Nova Institute has forecasted that the global production capacities of bioplastics in 2019 was estimated to be 2.11 million tonnes and could reach around 2.43 million tonnes in 2024 [105]. The analysis of techno-economic and life cycle impact by Beckstrom et al. [78] revealed that bioplastics could be sold for as low as $970 per tonne^−1^ and simultaneously reduce the emission of greenhouse gas by 67–116%. It can be said that the potential market for bioplastics is expected to rise and eventually replace the conventional plastics. The sustainable bioplastic materials are currently under development and it is essential to explore innovative materials or polymers from renewable sources for the sustainable manufacturing of bio-based and biodegradable plastics. This is because although some types of bioplastics are bio-based, they are not fully biodegradable and may bring a serious negative environmental impact. The materials used for manufacturing bioplastics must be biodegradable and preferably made from natural renewable resources like biomass, plants, waste resources, microalgae and bacteria [110]. These renewable materials must not compete with traditional sources used as foods while being able to reduce the use of non-renewable resources in the long term. On top of that, bioplastics produced must be environmentally and consumer-friendly, such as being free of odor throughout its lifetime. These types of bioplastics will have excellent thermal properties that are suitable as packaging material in food and beverage industry.

Algae are the promising candidate to synthesize polymer for sustainable bioplastics production and other co-products through biorefinery concept [[74], [111]]. The synthesis of consumer-grade bioplastics based on the monomers or polymers derived from algae waste residues is also feasible, solving the waste disposal problems [106]. This circular economy strengthens the eco-efficiency of bioplastics production processes and the production of other byproducts, such as fuel, nutraceuticals, pharmaceuticals and cosmetics that can be applied in different fields [[100], [112]]. Torres et al. [113] demonstrated the use of residual microalgae biomass from biodiesel production process and PBAT to synthesize biocomposites. In the work, plasticization improved the mechanical properties, tensile modulus and elongation of the biocomposites with the aid of 20% residual microalgae biomass and PBAT. The end products will decompose in the soil and was suggested to be used as agricultural films. These findings will encourage the industries to invest in research and development (R&D) of bioplastics through cooperation with academia to enhance the quality of algae-based bioplastics, industrialize new processes for bioplastics production and improve properties of bioplastics [114]. For instance, odor is one of the main consideration for the manufacturing of bioplastics with microalgae biomass as it may affect customer perception and its application as the packaging materials for food, drinks or other consumer products.

Furthermore, there is another concern regarding the policy and certification on the use of bioplastics. The certification process is a complex, time-consuming and tedious process because all the tests conducted involve the same material and is done by the same testing laboratory, so that there is no deviation of laboratory results from the actual conditions. Currently, there are no standard policies and specifications regarding the quality of bioplastics, although there are several certification systems for compostability of bioplastics [107,108]. According to European Bioplastics [109], one of the certification tests of bioplastics is EN 13432 (compostability test) which is to ensure that the product and its other components are compostable or can be industrially composted. Another certification test, EN 13432/EN 14995, comprises of chemical tests for heavy metals, biodegradability in controlled composting conditions, disintegration test, practical test of compostability in a semi-industrial (or industrial) composting facility, ecotoxicity test and others. These standard guidelines, policy, legislation and certification should be developed and recognized globally to ensure consistency in the usage of bioplastics or waste management, and to provide a structured procedure with low variation for quality and safety assurance.

In general, the increasing growth and business demand of the bioplastic industry in the future require discovery and developments of new biodegradable materials or polymers. This will lead to an increase in the production efficiency, while solving the plastic pollution and exploring the new opportunities of bioplastics in terms of quality and quantity of the bio-based and biodegradable bioplastics. The use of algae to manufacture bioplastics has shown a great potential in reducing the production cost, and it is a promising source and pathway to the bio-based and biodegradable bioplastics.

## 6 Conclusions

The extensive exploitation of fossil fuels and the increased global disposal of non-biodegradable conventional plastics in an uncontrollable manner are urging researchers to develop efficient methods to biodegrade plastic and alternative materials to substitute plastics, in order to mitigate marine plastic pollution. This is because marine plastic pollution can affect and endanger the marine life and their habitats, causing the extinction of certain aquatic species. Interestingly, algae can colonize on plastic surface and secrete enzymes to break down the plastics, using the plastic polymers as the carbon source for their energy and growth. Employing microalgae for plastic biodegradation provides several advantages compared to bacterial systems, hence it is presented as a potential solution. Bioplastics produced using microalgae are inexpensive and environmentally safe to substitute conventional plastics. However, the research on the algae-based bioplastics are still in the experimental or infancy stage and infeasible to be commercialized at industrial scale, making the advancement of technology and continual R&D in bioplastics significant. With further exploration of algal role in bioplastics degradations, a large portion of bioplastics can be produced from algae biomass sustainably in the near future.

## Declaration of competing interest

The authors declare that they have no known competing financial interests or personal relationships that could have appeared to influence the work reported in this paper.

## Abbreviations

BPA: Bisphenol A
EG: Ethylene glycol
GMO: Genetically modified organism
HDPE: High-density polyethylene
LDPE: Low-density polyethylene
MHETase: Mono(2-hydroxyethyl) terephthalic acid hydrolase
PBAT: Polybutylene adipate terephthalate
PE: Polyethylene
PET: Polyethylene terephthalate
PETase: PET hydrolase
PETG: Polyethylene terephthalate glycol
PHA: Polyhydroxyalkanoate
PHB: Polyhydroxybutyrate
PLA: Polylactic acid
PP: Polypropylene
PS: Polystyrene
PTT: Polytrimethylene terephthalate
PUFA: Polyunsaturated fatty acids
PVA: Polyvinyl alcohol
PVC: Polyvinyl chloride
R&D: Research and development
TPA: Terephthalic acid
TPC-ET: Polyester elastomers
